# Congenital Lobar Emphysema Presenting as Respiratory Distress in a Newborn

**DOI:** 10.7759/cureus.40545

**Published:** 2023-06-16

**Authors:** Sampa Choudhury, Suparna Dubey, Ritesh Kumar

**Affiliations:** 1 Pathology, Andaman and Nicobar Islands Institute of Medical Sciences (ANIIMS), Port Blair, IND; 2 Surgery, Andaman and Nicobar Islands Institute of Medical Sciences (ANIIMS), Port Blair, IND

**Keywords:** newborn, lung, hypoplasia, emphysema, congenital, cartilage

## Abstract

Congenital lobar emphysema (CLE) is a rare developmental malformation that presents as neonatal respiratory distress and can be successfully managed with prompt intervention. Hyperinflation of the affected lobe with mediastinal shift is the characteristic radiological finding. However, the etiology mostly remains unknown. Here, we report a case of CLE that microscopically revealed bronchial cartilaginous hypoplasia as an underlying cause of this malformation.

## Introduction

Congenital lobar emphysema (CLE), also known as congenital alveolar overdistension, congenital large hyperlucent lobe, and congenital lobar overinflation, is a rare developmental malformation of the lower respiratory tract that poses a diagnostic and therapeutic dilemma due to its wide range of presentations. CLE is characterized by respiratory distress due to the over-expansion of one or more pulmonary lobes, with compression of surrounding lung parenchyma, in the absence of tissue destruction [1]. The estimated incidence of CLE among live births is one in 20,000-30,000, with male predominance. It is common in infants and extremely rare among adults [2]. Here, we report a case of CLE in a newborn who presented with respiratory distress immediately after birth.

## Case presentation

**Figure 1:**
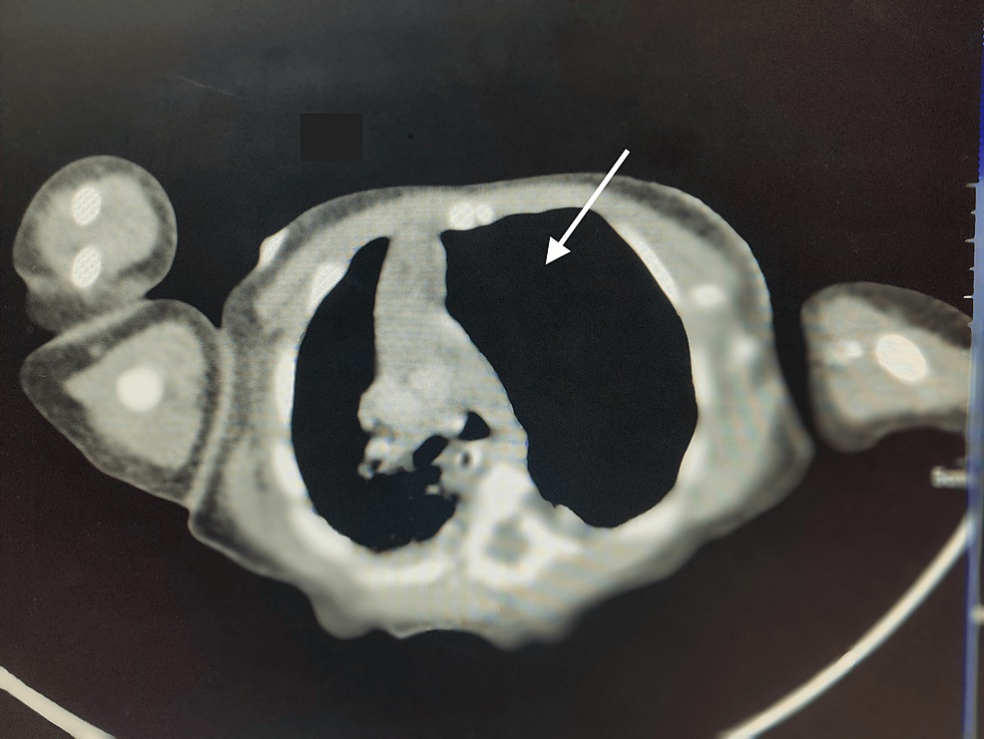
CT scan showing hyperinflation of the affected lobe with mediastinal shift (white arrow). CT: computed tomography.

A 2.75 kg term baby, delivered through cesarean section, was admitted to the neonatal intensive care unit (NICU) with findings of respiratory distress immediately after birth. Oxygen saturation of 98% was observed. She had tachypnea, tachycardia, and retractions. A provisional clinical diagnosis of pneumonia was considered, and the baby was started on antibiotics. On the third day, respiratory distress started improving, and a chest x-ray revealed a radiolucent zone in the left side of the chest. A clinical diagnosis of pneumothorax was made and an intercostal drain (ICD) was inserted, which led to decreased, but persistent respiratory distress. On day 13, the baby was sent for a computed tomography (CT) scan as respiratory distress was persistent and progressing even after ICD. The CT report showed hyperinflation of the left upper lobe with mediastinal shift, which led to suspicion of CLE (Figure 1). In view of worsening symptoms and a severe mediastinal shift, the baby was planned for surgical management. Her complete hemogram, kidney function test and echocardiography were within normal limit.

**Figure 2:**
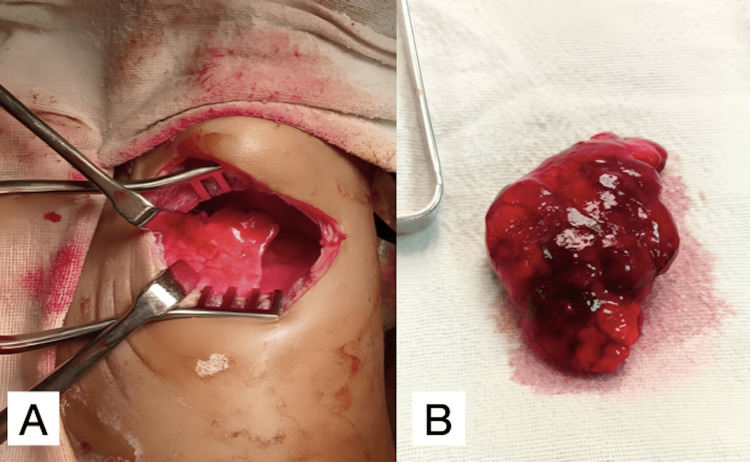
Intra-operative findings (A) open thoracotomy and (B) resected left upper lobe lung.

On the 14th day, she had undergone an open thoracotomy and left upper lobectomy (Figures 2A, 2B), and the specimen was sent for histopathological examination. Grossly, the lobectomy specimen measured 6×4×2 cm with a congested external surface. The cut surface showed grayish-white spongy areas, from which white frothy material oozed out.

**Figure 3:**
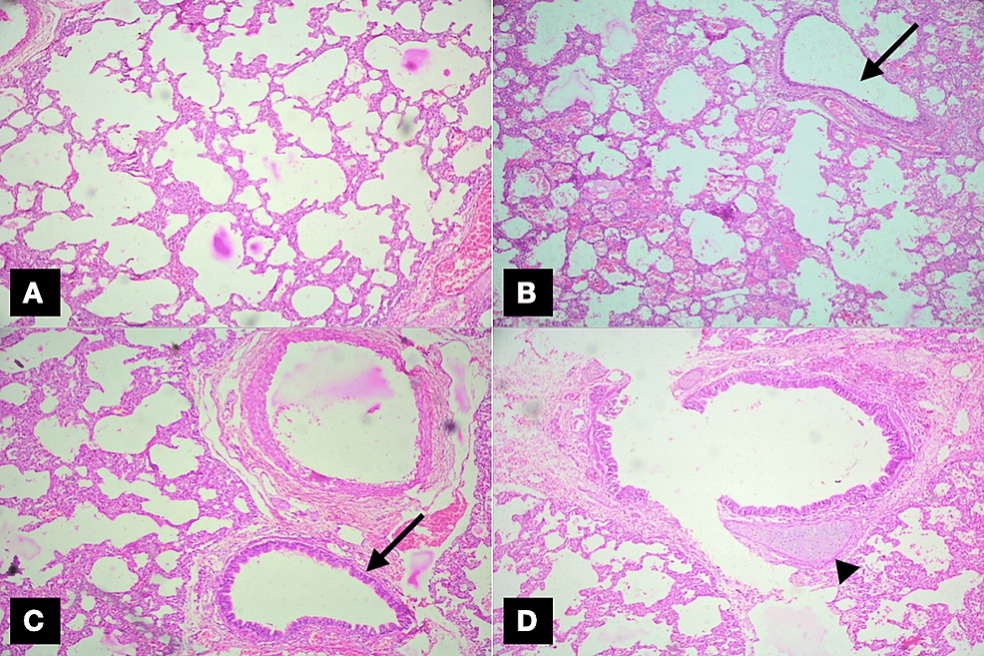
Microscopic findings. (A) Emphysematous changes of air spaces (H&E stain, 10×); (B) and (C) show focal areas of alveolar edema, hemorrhage, and bronchi without any cartilage plates (black arrow, H&E stain, 10×); (D) occasional bronchus with focal cartilage plate (arrowhead, H&E stain, 40×).

Microscopic examination revealed emphysematous changes of the air spaces, with focal areas of alveolar edema and hemorrhage. Very occasionally, bronchi showed cartilaginous plates (Figures 3A-3D). On the correlation of clinical, radiological, and pathological findings, a final diagnosis of congenital lobar emphysema was made. The baby has improved after the lobectomy without any signs of breathing difficulty. The ICD was removed on the third post-operative day, and the baby was discharged on the seventh post-operative day. In her follow-up appointments of six months and one year, she has reached the expected level of social, motor, language, and cognitive milestones for her age.

## Discussion

Congenital lobar emphysema is a rare congenital abnormality characterized by progressive overinflation of one or more lung lobes. It usually manifests within the first six months of birth with non-specific clinical features of dyspnea, tachypnea, wheezing, retractions, or pulmonary infections. Rarely, asymptomatic adults can be diagnosed incidentally [3,4]. A case of CLE in an adult presenting with pneumothorax has also been reported [5]. Left upper lobe (43%) involvement is more prevalent, followed by right middle lobe and right upper lobe [2]. Our patient presented at birth with the classical clinical findings and left lobe involvement.

The etiopathogenesis of CLE is not well established, and the etiology is unknown in 50% of cases. Bronchial cartilage anaplasia, hypoplasia, or dysplasia causes bronchial collapse and air trapping due to the ball valve mechanism in one-fourth of cases. Other infrequent causes are polyalveolar lobes and internal and external bronchial obstruction [5,6]. Emerging data suggest that minor transcription errors in the fibroblast growth factor-10 pathways, thyroid transcription factor-1 and Homeobox protein Nkx 2.1 may result in localized bronchial cartilage anomalies, leading to CLE, because these are essential for pulmonary branching morphogenesis [2].

Associated congenital anomalies involving the cardiovascular system (14-20%), followed by the renal, musculoskeletal, and gastrointestinal systems, are well documented. Clinical manifestations of other anomalies can sometimes be so prominent that CLE is easily overlooked [2,7]. Preoperative echocardiography, contrast-enhanced CT scan of the chest, renal function test, and other investigations are recommended to rule out any associated congenital anomalies [4,6].

Imaging studies can confirm the diagnosis of CLE before surgery. Overinflation of the affected lobe(s) with or without pulmonary vessel anomalies, atelectasis of the adjacent lobes, and mediastinal shift to the contralateral side are typical findings [3,8,9]. A ventilation-perfusion scan is helpful to detect ventilation-perfusion mismatches. Ultrasonography is useful for prenatal diagnosis but is frequently missed and eventually diagnosed postnatally due to respiratory distress [4,10]. Recently, magnetic resonance imaging (MRI) has gained popularity for prenatal diagnosis because of its better tissue contrast properties [2].

Differential diagnoses of congenital lobar emphysema include congenital cystic airway malformations, pneumothorax, pneumatocele, bronchogenic cyst, pneumonia, lung hypoplasia, opposite lung hyperinflation due to atelectasis, mucous plug or foreign body aspiration [2,8].

In older patients and conservatively managed patients, pre-operative bronchoscopy is very useful for detecting bronchomalacia, anatomical variations, endobronchial obstruction, and removing mucous plugs or foreign bodies. However, in newborns with respiratory distress, bronchoscopy should be performed with caution [1,2].

Surgical lobectomy is the most widely used treatment option, with excellent results; however, medical management can be considered for asymptomatic or mildly symptomatic patients [6,7]. A few surgeons have also advocated for segmental lung resection rather than lobectomies [10]. To minimize the complications, surgery must be performed as early as possible. Some authors have reported that the thoracoscopic approach is safer and more effective in children than the open method, but there is insufficient data to support this claim [6]. In our case, the baby was planned for surgical management immediately after the chest CT report, in view of the severe mediastinal shift and worsening respiratory symptoms.

## Conclusions

This is a case of a term female born with respiratory distress who was initially misdiagnosed as pneumonia and pneumothorax and was treated conservatively. Later on, the characteristic findings of CLE on chest CT prompted an immediate surgical intervention. Hence, congenital anomalies should be taken into consideration while dealing with newborns presenting with respiratory symptoms in order to minimize unnecessary delays in proper management and alleviate the patient’s suffering. Symptomatic CLE in a newborn is a medical emergency that requires immediate attention. It is noteworthy to remember that once diagnosed, a search for associated congenital anomalies is warranted.
